# Climate-Driven Changes in Potential Suitability and Spatial Co-Occurrence Across the Pine Wilt Disease Complex

**DOI:** 10.3390/insects17080819

**Published:** 2026-08-06

**Authors:** Xianheng Ouyang, Hongbo Duan, Zhikuan Cao, Yuntian Liu, Fanrui Ge, Peng Nie, Shihao Wang, Tayyab Shaheen, Qiaoyun Sun

**Affiliations:** 1 College of Forestry, Northwest A&F University, Yangling 712100, China; 2 College of Plant Protection, Northwest A&F University, Yangling 712100, China; 3 College of Natural Resources and Environment, South China Agricultural University, Guangzhou 510642, China; 4 Ankang Baihe County Forestry Bureau, Ankang 725800, China; 5 College of Agriculture and Biotechnology, Zhejiang University, Hangzhou 310058, China; 6 School of Architecture and Urban Planning, Shenzhen University, Shenzhen 518060, China

**Keywords:** climate change, pine wilt disease, ensemble species distribution models, potential suitability, spatial co-occurrence, *Monochamus alternatus*

## Abstract

Climate change may redistribute forest pests, pathogens, natural enemies, and host trees, yet these components are still often projected independently. We used ensemble species distribution models (SDMs) to map potential climatic suitability for *Monochamus alternatus*, *Dastarcus helophoroides*, *Scleroderma guani*, *Bursaphelenchus xylophilus*, and a genus-level *Pinus* host layer, and then summarized niche overlap, range overlap, and multispecies co-suitability under SSP1-2.6, SSP3-7.0, and SSP5-8.5 for the 2050s and 2090s. A structural equation model (SEM) fitted to thresholded binary layers was retained only as an exploratory description of conditional spatial associations. Because its inputs were suitability classifications rather than abundance, infection, parasitism, nematode load, or transmission data, neither arrow direction nor coefficient sign is interpreted causally. The strongest joint modeled suitability for the vector, pathogen, host, and at least one natural enemy occurred in East Asia, whereas *B. xylophilus* alone also showed potential climatic suitability in parts of the Americas and Africa. Importantly, the current model underpredicted the established occurrence of *B. xylophilus* in Portugal and western Spain, demonstrating that mapped unsuitable cells cannot be interpreted as confirmed absence. The genus-level *Pinus* layer similarly represents broad host availability rather than species-specific susceptibility. Because the future maps are based on averages across three general circulation models and the archived outputs do not permit retrospective estimation of inter-model variability, these projections should be treated as screening-level, scenario-conditioned summaries rather than uncertainty-bounded forecasts. The results identify priorities for surveillance and field validation, but they do not quantify disease incidence, interaction strength, or biological-control efficacy.

## Simple Summary

Pine wilt disease is a serious threat to pine forests and results from the combined effects of the pine wood nematode, insect vectors, host trees, and natural enemies. Climate change may alter where these organisms can survive, but their distributions may not shift together. We used multiple species distribution models to compare the present and future climatic suitability of the main vector *Monochamus alternatus*, the nematode *Bursaphelenchus xylophilus*, two natural enemies, and *Pinus* host trees. The strongest joint modeled suitability was concentrated in East Asia, particularly southeastern China and Japan, although the nematode alone also showed suitable climates in parts of the Americas and Africa. Natural enemies overlapped with the vector in some regions, identifying locations where biological control could be tested, but overlap does not guarantee effective suppression. The maps describe potential climatic suitability rather than confirmed occurrence or future disease incidence. They should therefore be combined with field surveillance, information on susceptible pine species, local vectors, introduction pathways, and biological-control performance when planning forest protection.

## 1. Introduction

Rapid climate change is reorganizing species distributions and, in parallel, reshaping the spatial template on which ecological interactions occur [1,2,3]. Rising temperatures, altered precipitation regimes, and more frequent climatic extremes can relax environmental constraints for some taxa while imposing stronger constraints on others, thereby redistributing biodiversity and reconfiguring pest–pathogen–host–natural enemy networks [4]. These processes are especially consequential for forest health because climate-driven range shifts can open invasion pathways, increase establishment probability, and modify the geographic match between invasive pests, pathogens, host plants, and biological control agents [5,6,7]. Understanding the spatial ecology of such multitrophic systems is therefore central to predicting the biological consequences of climate change.

Pine wilt disease provides a tractable but high-impact system in which climate, invasion biology, insect behavior, and pathogen transmission intersect. In East Asia, the Japanese pine sawyer *Monochamus alternatus* is a major vector of the pine wood nematode *Bursaphelenchus xylophilus*, the causal agent of pine wilt disease [8,9,10,11]. The epidemiology of the disease is tightly coupled to the vector life cycle. *M. alternatus* develops through egg, larval, pupal, and adult stages inside or on host trees; larvae feed in phloem and xylem galleries, overwinter in wood, and adults emerge seasonally as temperature accumulates [12,13,14]. Newly emerged adults are reproductively immature and conduct maturation feeding on the bark and phloem of current-year pine shoots, which is one of the primary pathways by which *B. xylophilus* enters healthy trees through feeding wounds [15,16]. Females subsequently select weakened, dying, dead, or freshly cut pine material for oviposition, linking vector reproduction to host stress, stand condition, and the availability of suitable breeding substrates [8,12].

Several biological traits make *M. alternatus* particularly important under climate change. Adult emergence phenology, maturation feeding intensity, reproductive timing, and larval development are temperature-sensitive, so warming can modify both the seasonal window of nematode transmission and the number of host patches exposed to infectious adults [12,17]. In addition, *M. alternatus* is not a passive occupant of climatically suitable cells. Flight-mill and field evidence indicate that adult *Monochamus* beetles can move from local tree-to-tree distances to kilometre-scale dispersal, and flight performance varies with sex, age, body size, and host condition [8,14]. This mobility allows the vector to bridge discontinuous host patches, exploit transient resources such as storm-damaged or recently felled wood, and potentially occupy climate-marginal patches over ecological timescales that are much shorter than those of host-tree range shifts.

Natural enemies add a further mechanistic layer to this disease complex. *Dastarcus helophoroides* is an ectoparasitic beetle that attacks late-instar larvae, pupae, and young adults of longhorn beetles, including *M. alternatus*, within wood galleries [18]. Its host-location process is not merely a response to the broad distribution of *M. alternatus*. *D. helophoroides* females lay eggs near host entrance holes, frass-extrusion holes, or tunnel walls; neonate larvae actively search for concealed hosts; and adults respond to host-associated frass, pine oleoresin monoterpenes, and visual cues associated with potential oviposition sites [18,19,20]. *Scleroderma guani*, by contrast, is a parasitoid wasp with a broader host range that can be mass-reared and released in augmentative biological-control programs [21,22]. Its females use innate and learned chemical cues, including frass-associated cues, to locate concealed *M. alternatus* larvae under bark [23]. These differences in host specificity, microhabitat use, cue ecology, and life-stage targeting mean that climate change may affect biological control not only through distributional overlaps but also through phenological and behavioral synchrony.

**Figure 1:**
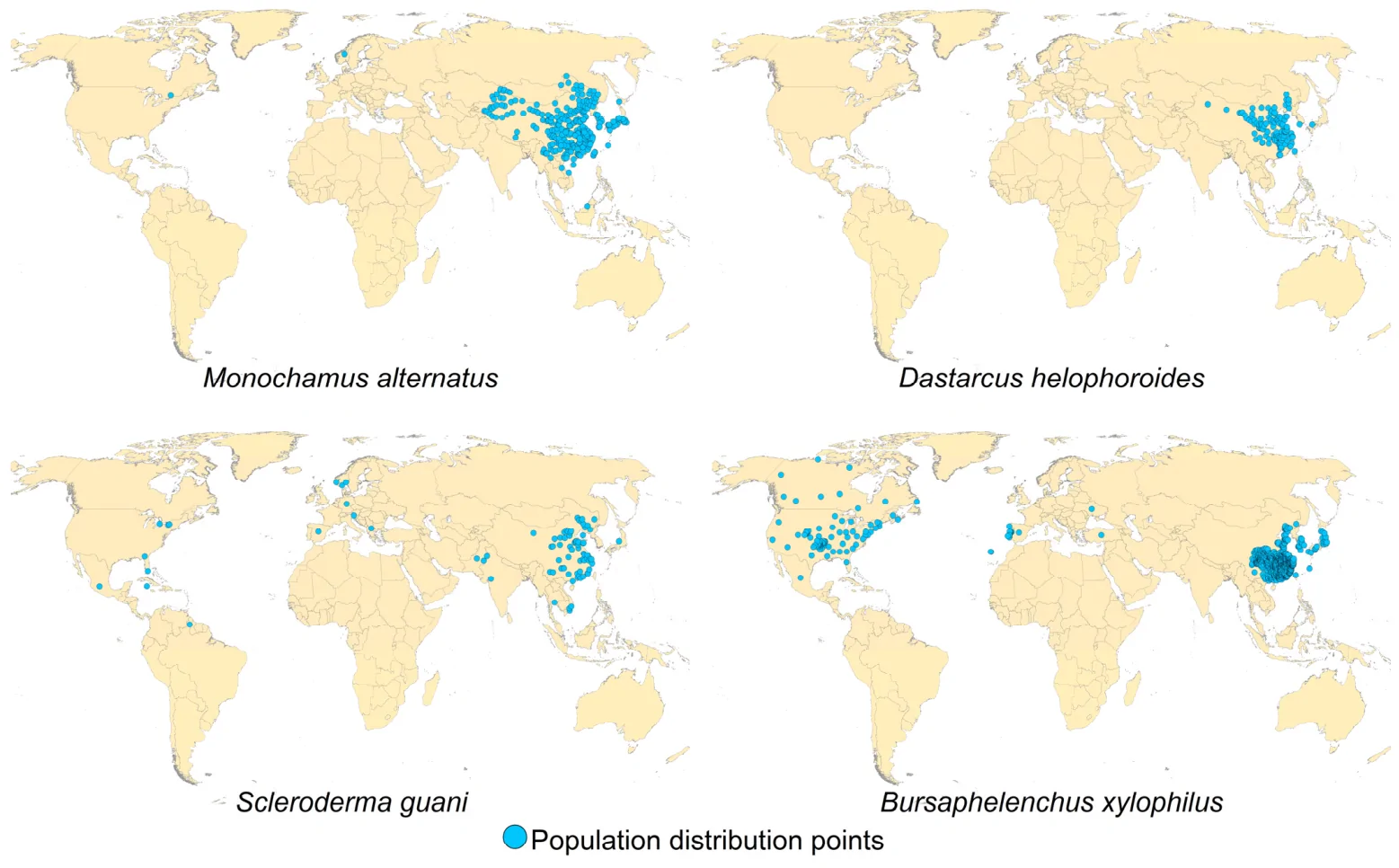
Global occurrence records used for SDM calibration for *Monochamus alternatus*, *Dastarcus helophoroides*, *Scleroderma guani*, and *Bursaphelenchus xylophilus*. Blue points indicate occurrence localities; differences in point density should not be interpreted as differences in abundance.

Occurrence records for the four individually mapped taxa spanned Asia, Europe, Africa, and the Americas, although sampling density was uneven and concentrated in East Asia (Figure 1). This geographic breadth motivated a global screening analysis, while the uneven coverage requires cautious interpretation of extrapolation and geographic sampling bias.

SDMs are designed to estimate taxon-specific potential climatic suitability from occurrence–environment relationships; they do not, by themselves, quantify multispecies interactions (Table S1) [24,25,26]. When independently fitted suitability surfaces are aligned on a common grid, niche, range-overlap, and richness summaries can identify where modeled climatic opportunity is shared. A path model can additionally describe conditional covariation among those derived layers, but it cannot validate the SDMs or convert spatial coincidence into a mechanistic interaction. In particular, directed coefficients estimated from thresholded binary indicators are not evidence of causal ecological effects, co-occurrence intensity, parasitism, infection, or transmission [27,28,29].

Here, the analytical sequence was explicit rather than a single coupled predictive model: we first fitted ensemble SDMs independently for each component, then quantified niche similarity, geographic range overlap, response curves, and multispecies co-suitability, and finally used an exploratory path analysis to describe conditional associations among the thresholded binary layers. We asked four questions: (i) where are current suitable habitats and overlap hotspots for the vector, pathogen, natural enemies, and host; (ii) how do these habitats shift under SSP1-2.6, SSP3-7.0, and SSP5-8.5 in the 2050s and 2090s; (iii) which climatic gradients structure the dominant modeled responses of the focal components; and (iv) what conditional spatial associations are recovered when the inputs are thresholded binary suitability layers. The inferential target is potential co-suitability at a broad geographic scale, not realized interaction strength, disease incidence, or biological-control performance. The complete analytical workflow is summarized in Figure S1.

## 2. Materials and Methods

### 2.1. Occurrence Records

Occurrence records for the focal pest, pathogen, and natural enemy taxa were compiled from major biodiversity repositories, including the Global Biodiversity Information Facility (GBIF, https://www.gbif.org/, accessed on 1 January 2025) and the Centre for Agriculture and Biosciences International (CABI, https://www.cabi.org/, accessed on 1 January 2025), together with peer-reviewed literature, technical reports, and other publicly available sources [8,23,30,31,32,33]. Records lacking coordinates but providing sufficiently detailed locality information were georeferenced using Google Maps (https://www.google.com/maps, accessed on 1 January 2025). The five modeled components comprised four individually mapped taxa (*M. alternatus*, *D. helophoroides*, *S. guani*, and *B. xylophilus*) and a genus-level *Pinus* host layer. The *Pinus* layer was intended to represent broad host availability at regional-to-continental scales; it does not represent species-specific susceptibility, stand condition, or local host stress. Standalone current and future maps are presented for the four non-host taxa, whereas *Pinus* is included in the overlap, multispecies richness, response-curve, and exploratory path analyses. Figure 1 shows the occurrence records used to calibrate the four individually mapped taxa.

### 2.2. Environmental Variables

Contemporary climate data (1970–2000) and future climate projections for the 2050s (2041–2060) and 2090s (2081–2100) were obtained from WorldClim at 2.5 arc-minute resolution [34,35]. The initial candidate set comprised the 19 WorldClim bioclimatic variables, elevation, and the human footprint index; definitions and sources are listed in Supplementary Table S2. Future projections were represented by SSP1-2.6, SSP3-7.0, and SSP5-8.5 [36]. Future predictors were averaged across three general circulation models (GCMs): ACCESS-CM2, BCC-CSM2-MR, and CMCC-ESM2 [37]. The maps therefore represent three-GCM mean conditions and do not display inter-model variability. Taxon-specific screening was used to reduce collinearity before model fitting, but the archived project materials do not retain the complete taxon-level final-variable and variance inflation factor table. This incomplete record limits exact computational reconstruction and is acknowledged in Section 4.6.

### 2.3. Species Distribution Models

Ecological niche models were developed for the four individually mapped taxa and the genus-level *Pinus* host layer under current and future climates using the biomod2 (v. 4.2.5) package in *R* [38]. We used an ensemble framework because comparing multiple modeling approaches reduces dependence on the assumptions of any single approach and allows model-specific variation in projected suitability to be evaluated [39,40,41].

The candidate set comprised 12 modeling approaches implemented through the biomod2 framework; these were approaches within a common workflow, not 12 separate software packages. We grouped them by model family: regression and smoothing methods (GLM, GAM, and MARS); classification and discriminant methods (CTA and FDA); tree-based ensemble and boosting methods (GBM, RF, and XGBoost); an artificial neural network (ANN); maximum-entropy methods (MaxEnt and the *maxnet* implementation, MAXNET); and the species range envelope (SRE). These families differ in how they represent nonlinear responses, interactions, and response-shape complexity, so comparing them reduced dependence on any single modeling assumption. Full names and other abbreviations are provided in Supplementary Table S1.

Model settings were optimized using the tuning function in biomod2. For each target component, 75% of occurrence records were used for calibration and 25% for evaluation; presence and pseudo-absence observations were weighted to maintain balance. Five repeated splits were used, yielding 60 model-by-repeat fits per component and 300 fits across all five components before ensemble filtering and summarization.

Individual models were evaluated with the true skill statistic (TSS), and only models with evaluation TSS > 0.7 were retained for ensemble construction [42]. For binary classification, we used the evaluation-data threshold that maximized sensitivity + specificity − 1. Ensemble outputs were summarized using the archived integration outputs (mean, coefficient of variation, confidence interval, median, consensus mean, and weighted mean). Continuous suitability outputs were classified into unsuitable, low-, moderate-, and high-suitability classes for mapping and converted to binary suitable/unsuitable layers for overlap and exploratory path analyses. Because pseudo-absence selection and the numerical taxon-specific binary cut-offs can influence broad-scale projections, overlap and SEM results were interpreted as comparative, threshold-dependent spatial summaries rather than exact forecasts.

### 2.4. Ecological Niche Comparison

Niche similarity and range overlap among *M. alternatus*, *D. helophoroides*, *S. guani*, *B. xylophilus*, and *Pinus* were quantified in *ENMTools v1.3* [43,44,45]. We used the Hellinger-based similarity index (I) to estimate pairwise niche overlap because it provides a bounded measure of similarity in environmental space and is relatively insensitive to extreme values [46].

Geographic range overlap was calculated from the binary suitability maps to quantify the degree of spatial coincidence among potentially suitable habitats. Both indices range from 0 to 1, with higher values indicating stronger overlap in environmental space or geographic distribution. Evaluating niche similarity and range overlap together allowed us to distinguish taxa that share similar climatic envelopes from taxa that actually coincide geographically under current or future climates.

### 2.5. Exploratory Path Analysis of Binary Spatial Associations

SEM syntax was implemented in *R* using the *lavaan* package to summarize conditional associations among the five binary suitability layers [47]. The grid cell was the analytical unit, and the model was retained as an exploratory path analysis rather than a mechanistic population model. Arrow direction in Figure S2 records the regression equations specified in the fitted model; it does not establish temporal or biological causation. An undirected covariance network would represent a different statistical model. Each input remained coded as 0/1 (unsuitable/suitable or absence/presence) and therefore represented a thresholded classification, not continuous suitability, abundance, population density, parasitism rate, nematode load, infection prevalence, or transmission intensity. The fitted coefficients can describe conditional covariation among classifications, but they cannot measure co-occurrence intensity or ecological interaction strength. Crucially, no direct observations of interspecific interactions entered the path model; all five variables were thresholded binary layers derived from independently fitted SDMs. Accordingly, this path analysis should be regarded as exploratory and non-reproducible from the surviving project archive.

No z-standardization was applied to the binary taxon layers before the exploratory path analysis; they remained coded as 0 and 1. The term standardized path coefficient refers to the standardized solution reported by *lavaan*, not to standardized biological abundance or interaction data. Model fit was summarized using CFI, TLI, SRMR, AIC, BIC, and the chi-square goodness-of-fit test [27,48,49]. These values are internal diagnostics of the specified covariance structure. Given the binary inputs, strong covariance, and very small model degrees of freedom, they are not evidence that the model is causally or biologically adequate.

### 2.6. Interpretation of Correlative Projections and Dispersal

In this study, SDMs, spatial overlays, and SEM served distinct, sequential purposes. First, SDMs were fitted independently for each focal component to estimate potential climatic suitability. Second, the thresholded suitability layers were overlaid to describe geographic co-suitability and range overlap. Third, SEM was used only as a secondary exploratory analysis of conditional statistical associations among the binary suitability layers at the grid-cell level. The SEM neither fed back into SDM fitting nor validated or improved the SDM predictions, and it was not used to infer causal ecological interactions, infection processes, parasitism, transmission, or biological-control efficacy.

Accordingly, all SDM, overlap, and SEM outputs were interpreted as screening-level summaries of potential climatic suitability, spatial co-suitability, and conditional spatial associations, rather than as evidence of realized occupancy or realized interaction strength. This distinction is especially important for mobile insects. Although SDMs relate occurrence records to environmental predictors, realized distributions may depart from modeled climatic suitability when dispersal, host availability, disturbance, or human-mediated transport are important. Adults of *M. alternatus* can disperse from local tree-to-tree distances to kilometre scales and may reach newly available host patches more rapidly than host trees can shift their ranges [8,14,50]. Consequently, beetles may occur in host-containing patches that are classified as climatically marginal by the SDMs, particularly after disturbance, sanitation cutting, wood transport, or episodic warming. These limitations guided our interpretation of future overlap patterns and management implications.

## 3. Results

### 3.1. Model Evaluation

Across the five focal components, the 12 candidate modeling approaches showed heterogeneous performance (Supplementary Table S3). Ensemble models performed consistently well, with AUC ranging from 0.89 to 0.99, TSS from 0.80 to 0.97, and KAPPA from 0.81 to 0.90. These evaluation statistics do not quantify geographic transferability, independent field validation, or uncertainty among GCMs.

### 3.2. Current Potential Geographic Distribution and Overlap

**Figure 2:**
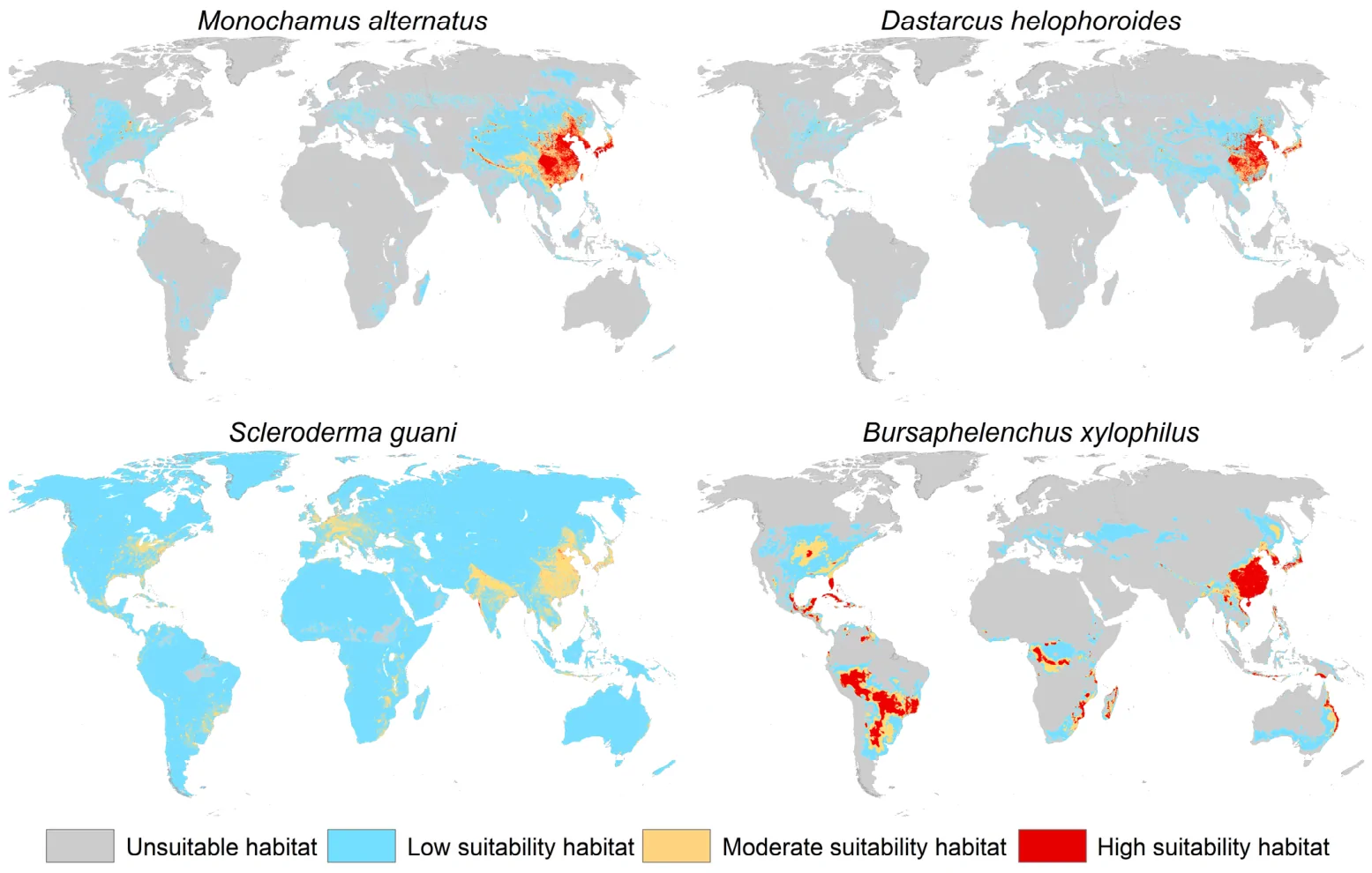
Current modeled potential geographic distributions of *Monochamus alternatus*, *Dastarcus helophoroides*, *Scleroderma guani*, *and Bursaphelenchus xylophilus*. Suitability classes were derived from thresholded ensemble forecasts and represent modeled potential suitability rather than confirmed presence or absence.

Current SDMs indicated that *M. alternatus*, *D. helophoroides*, and *B. xylophilus* have important suitability cores in East Asia, especially eastern and southeastern China, the Korean Peninsula, and Japan, whereas *S. guani* showed a broad low-suitability envelope across parts of Asia, Europe, Africa, and the Americas but a much smaller high-suitability core (Figure 2). However, the *B. xylophilus* model classified much of Portugal and western Spain below the suitability threshold despite established occurrence in the Iberian Peninsula and occurrence records shown in Figure 1. This is a false-negative region in the present model; the mapped unsuitable class must not be interpreted as confirmed absence or zero establishment risk.

*S. guani* displayed a different pattern: broad low-to-moderate suitability occurred across many temperate and subtropical regions, but high suitability was limited. *B. xylophilus* showed potential climatic suitability in East Asia and in additional warm, humid regions of the Americas, Africa, and island systems. These single-taxon projections identify environmental opportunity for the nematode, not locations where pine wilt disease is predicted to occur. In the present five-component overlay, the strongest joint modeled suitability of *B. xylophilus*, *M. alternatus*, *Pinus*, and at least one natural enemy was concentrated in East Asia.

**Figure 3:**
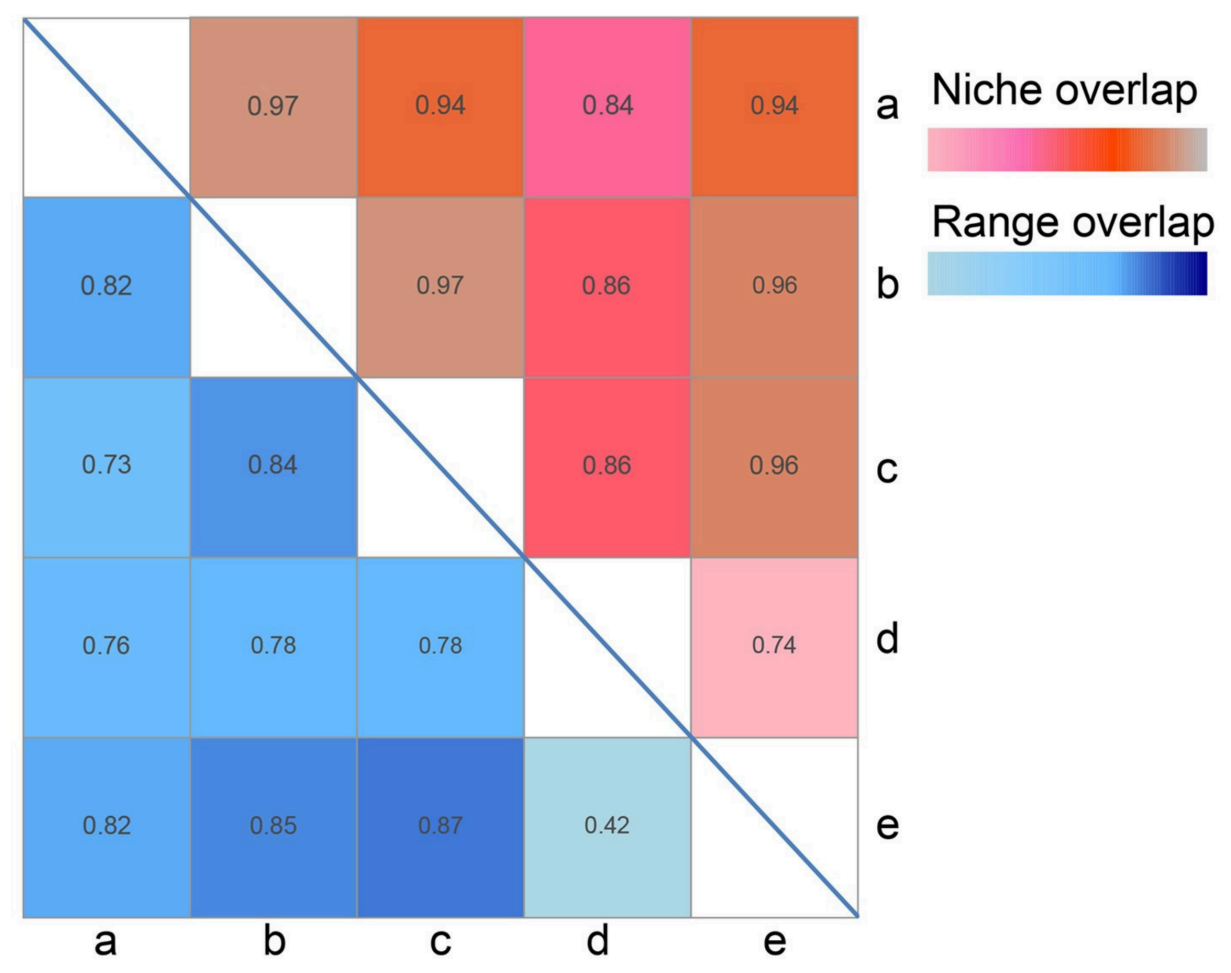
Pairwise niche similarity and geographic range overlap among the five focal components. Upper triangle: Hellinger-based niche overlap. Lower triangle: range overlap based on thresholded binary suitable habitat. (**a**) *Monochamus alternatus*; (**b**) *Dastarcus helophoroides*; (**c**) *Scleroderma guani*; (**d**) *Bursaphelenchus xylophilus*; and (**e**) *Pinus*.

Pairwise niche and range overlap among the five components were substantial but uneven (Figure 3). Climatic niche overlap was highest between *M. alternatus* and *D. helophoroides* (I = 0.97) and between *D. helophoroides* and *S. guani* (I = 0.97), followed by high values for *D. helophoroides*-*Pinus* and *S. guani*-*Pinus* (both 0.96). These values indicate that several vector, natural-enemy, and host components occupy closely aligned climatic space.

Geographic range overlap also showed strong but not identical patterns. *M. alternatus* overlapped strongly with *D. helophoroides* (0.82), *S. guani* (0.73), and *B. xylophilus* (0.76). The highest range-overlap values involving *Pinus* occurred with *S. guani* (0.87), *D. helophoroides* (0.85), and *M. alternatus* (0.82), whereas *B. xylophilus* and *Pinus* had lower range overlap (0.42) despite moderate niche similarity (I = 0.74). This contrast indicates that climatic compatibility does not necessarily translate into uniform geographic coincidence.

The high overlap between *M. alternatus* and *D. helophoroides* is more specific than generic host tracking. It identifies landscapes in which the vector climate envelope and the natural enemy climate envelope coincide, and where concealed larval and pupal stages of longhorn beetles may be available in pine wood galleries. However, overlap alone cannot estimate parasitism rate, suppression strength, or local biological-control success.

### 3.3. Potential Geographic Distributions Under Future Climate Scenarios

**Figure 4:**
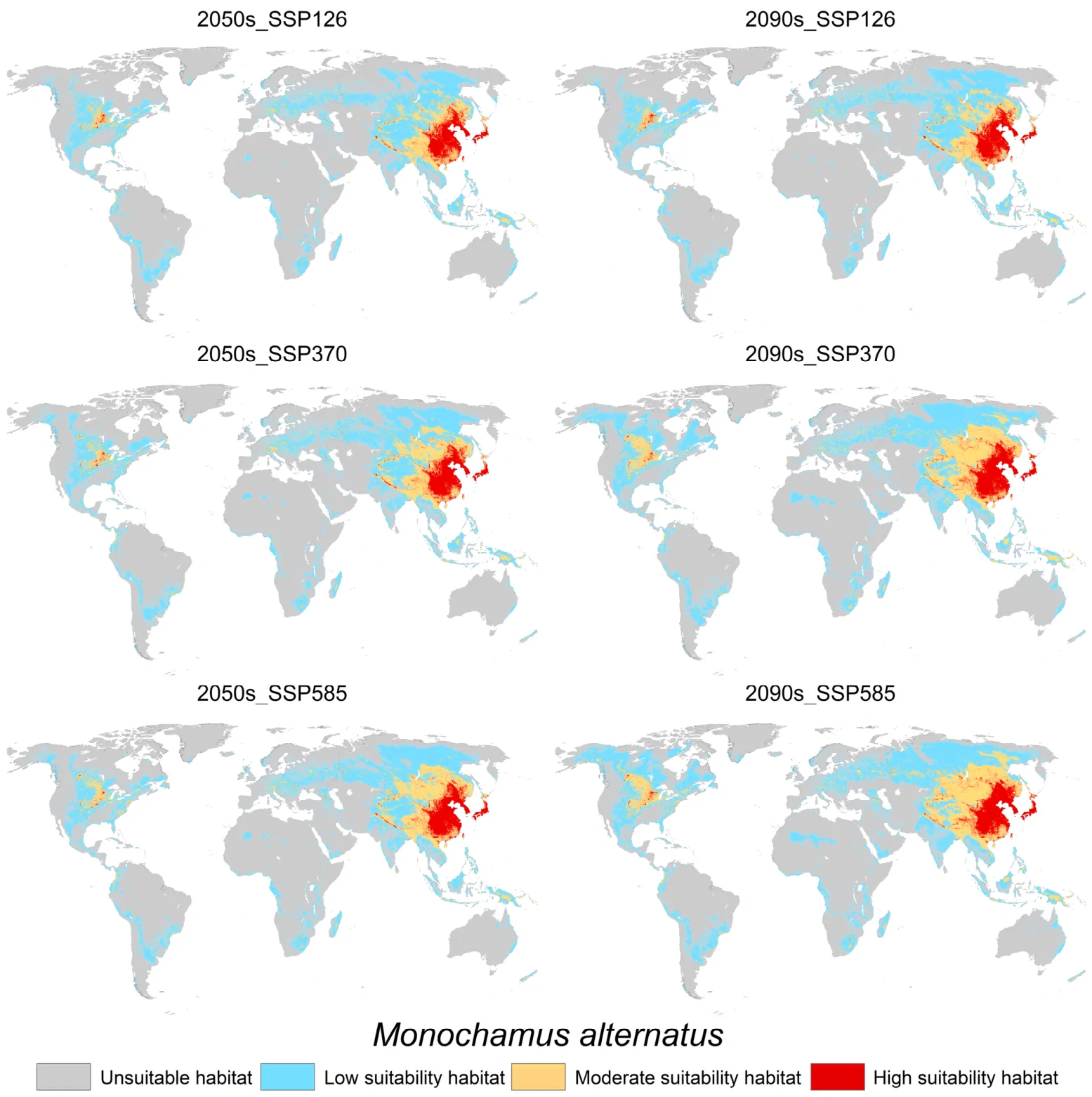
Projected future potential geographic distribution of *Monochamus alternatus* under SSP scenarios for the 2050s and 2090s.

**Figure 5:**
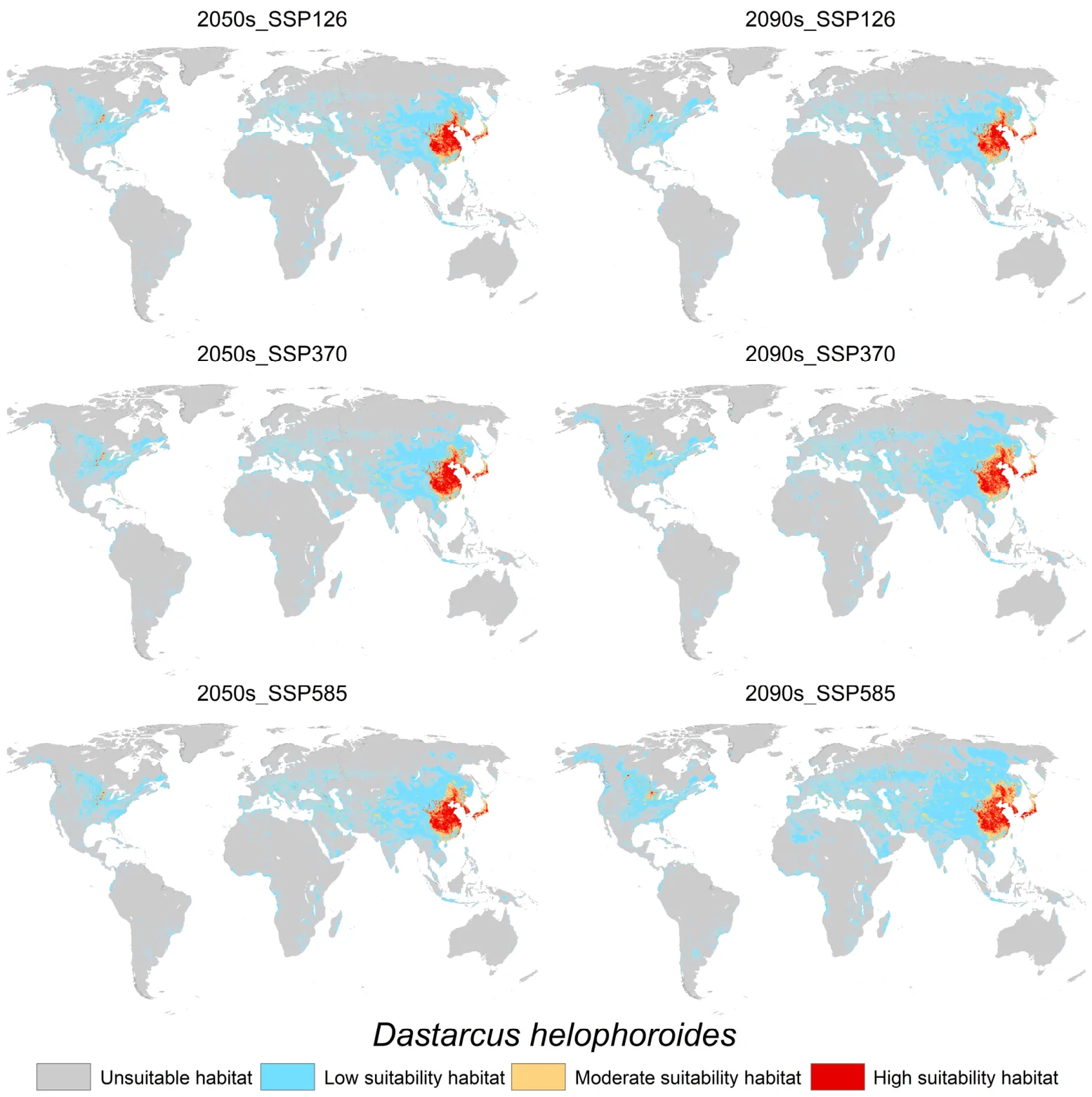
Projected future potential geographic distribution of *Dastarcus helophoroides* under SSP scenarios for the 2050s and 2090s.

**Figure 6:**
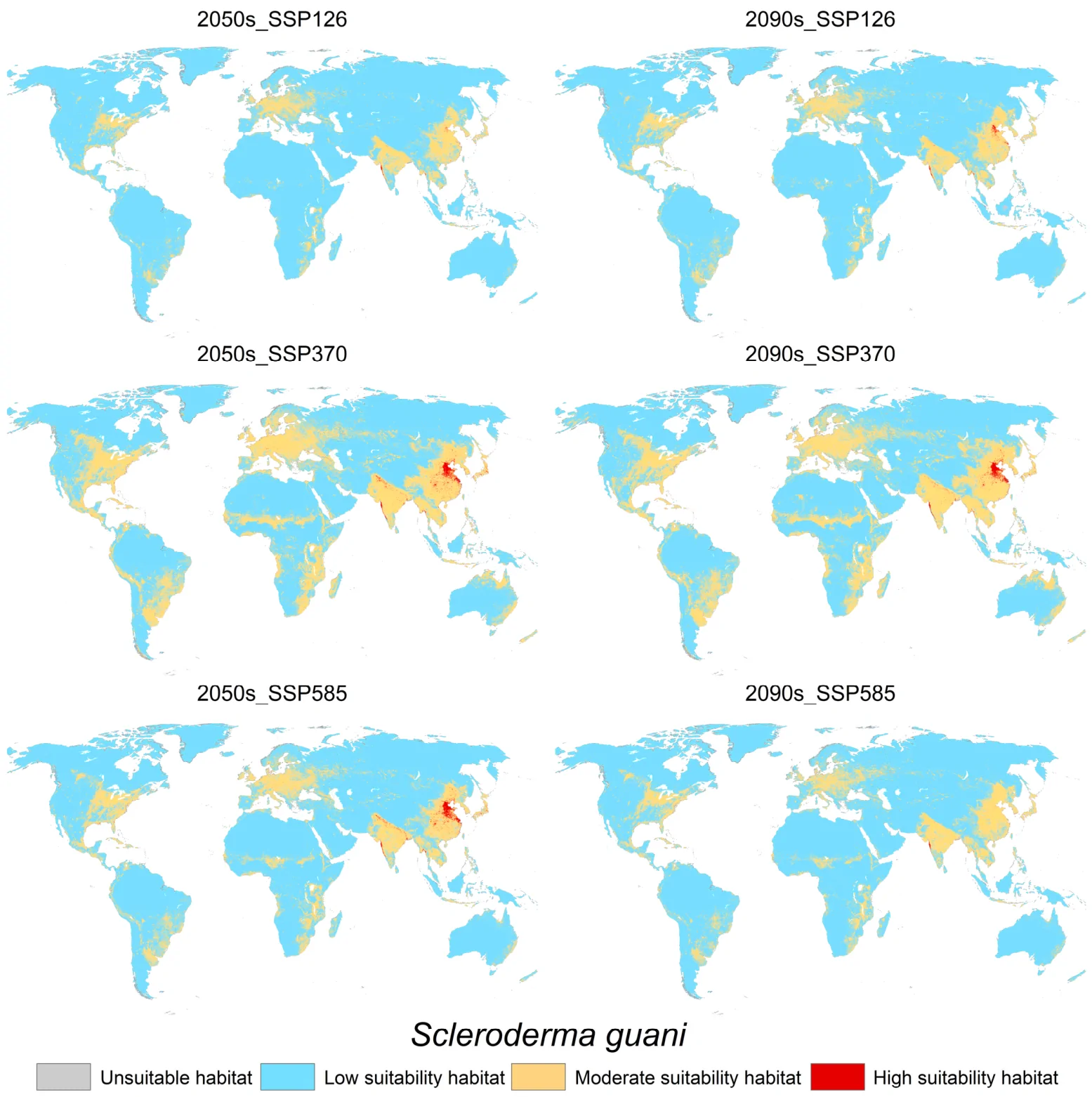
Projected future potential geographic distribution of *Scleroderma guani* under SSP scenarios for the 2050s and 2090s.

**Figure 7:**
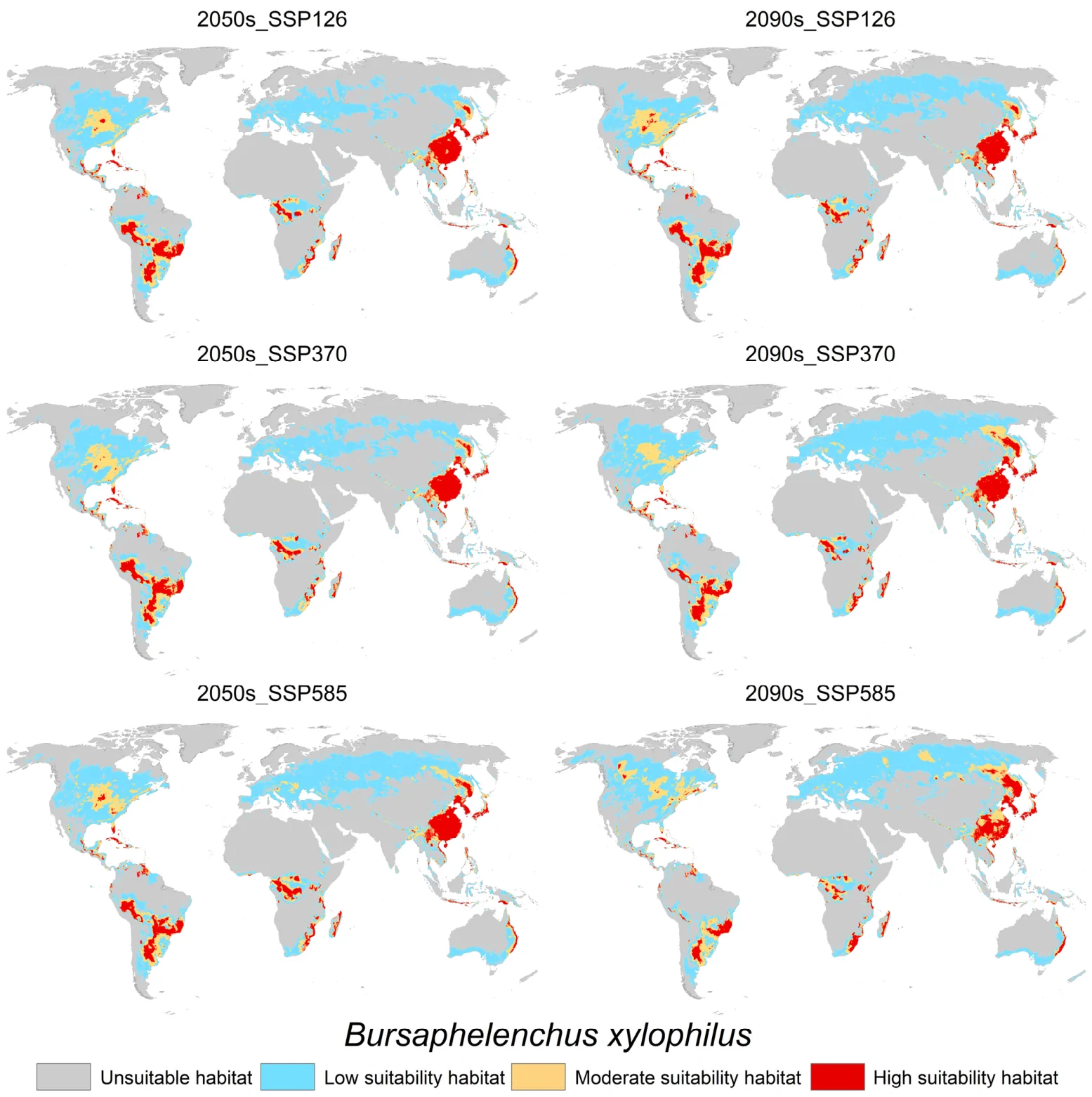
Projected future potential geographic distribution of *Bursaphelenchus xylophilus* under SSP scenarios for the 2050s and 2090s.

Future projections showed changes in modeled suitability across the pine wilt disease complex, with East Asia remaining the most persistent area of high joint modeled suitability (Figure 4, Figure 5, Figure 6 and Figure 7; Supplementary Tables S4–S7). For *M. alternatus*, moderate- and high-suitability areas increased across most scenarios, while unsuitable areas declined from 10,690.83 × 10^4^ km^2^ in the 2050s SSP1-2.6 scenario to 8881.19 × 10^4^ km^2^ in the 2090s SSP5-8.5 scenario. High-suitability habitat increased from 335.56 to 476.55 × 10^4^ km^2^ over the same comparison, with the largest gains concentrated in southeastern China, eastern China, and Japan (Figure 4). All future maps are based on means across three GCMs; because inter-model dispersion was not retained in the archived outputs, apparent differences among SSPs should not be interpreted as uncertainty-bounded forecasts.

### 3.4. Effects of Climate Change on Potential Multispecies Richness

**Figure 8:**
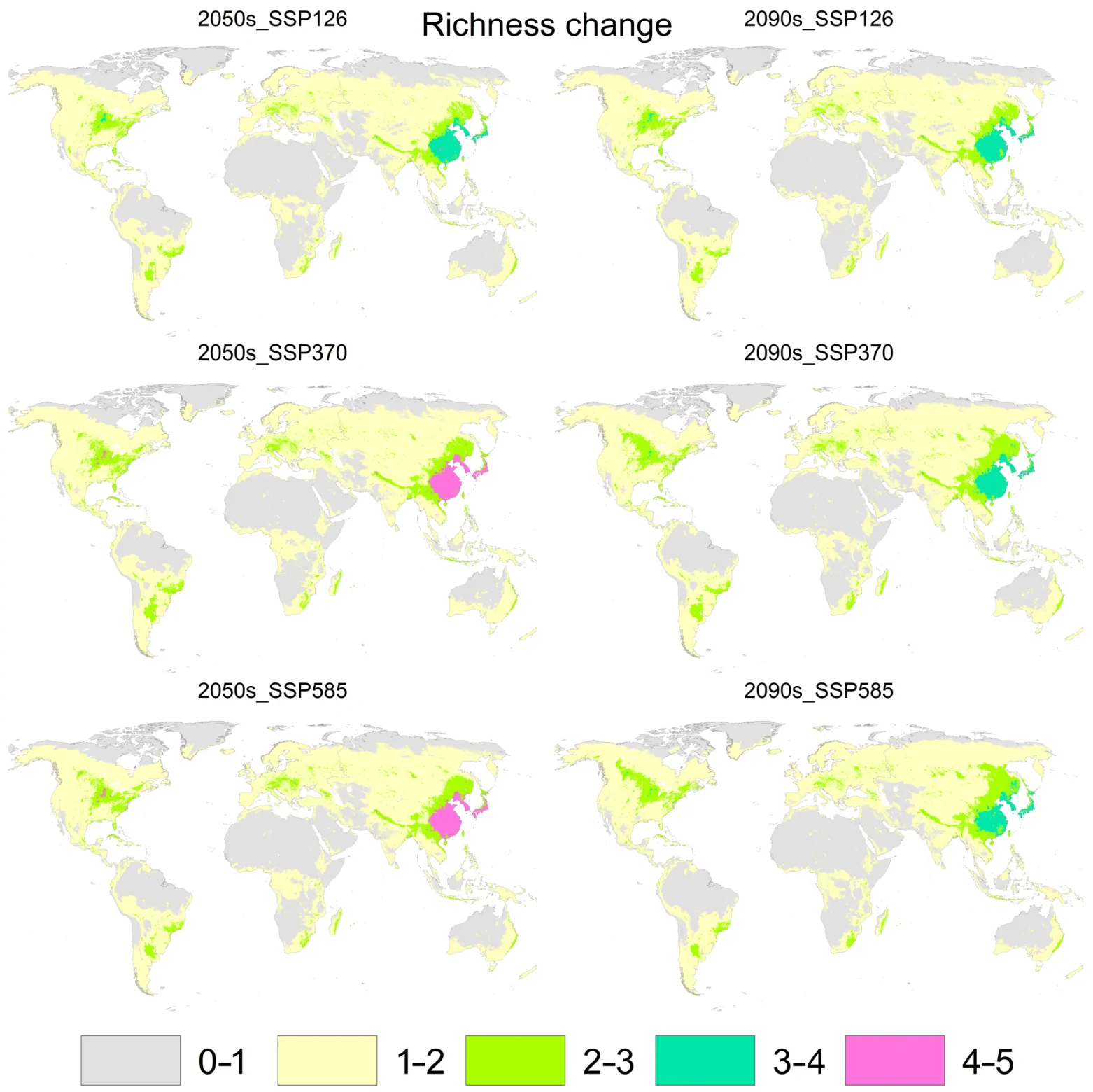
Future multispecies richness and spatial distribution of suitable habitat overlap under SSP scenarios. Classes represent the number of focal components with thresholded suitable habitat per grid cell.

Multispecies richness maps, expressed as the number of focal components with suitable habitat per grid cell, showed that low-richness classes (0–1 and 1–2 components) dominated high latitudes, deserts, and large parts of Africa and the Americas (Figure 8). Higher-richness classes (3–4 and 4–5 components) were concentrated mainly in climatically favorable regions of East Asia, Southeast Asia, and, to a lesser extent, parts of the Mediterranean and temperate Europe.

The highest richness class was most evident in eastern and southeastern China under several future scenarios, especially where *M. alternatus*, *B. xylophilus*, *Pinus*, and at least one natural enemy were projected to coincide. From the 2050s to the 2090s, areas supporting three or more components generally increased under all SSPs, while low-richness areas contracted in some temperate regions. These maps indicate a growing probability of multispecies encounters and shared climatic opportunity rather than a direct measure of disease incidence or biological-control efficacy.

### 3.5. Dominant Climatic Responses and Exploratory Path Associations

Response curves for the dominant predictors revealed both shared climatic filters and species-specific tolerances (Figure S3). For temperature seasonality (bio4), *M. alternatus* showed high suitability at intermediate values, with a broad response extending across much of the warm-temperate range. *D. helophoroides* and *B. xylophilus* showed partially overlapping but narrower optima, whereas *Pinus* retained comparatively broad suitability across a wider climatic window. These patterns suggest that the vector and one natural enemy share a strong climatic filter, but the host genus spans a wider environmental breadth.

The response to mean temperature of the coldest quarter (bio11) showed a stronger separation among components. *M. alternatus*, *D. helophoroides*, *S. guani*, and *B. xylophilus* generally had high suitability across cool-to-mild winter conditions, whereas *Pinus* showed a distinct peak under warmer cold-quarter temperatures. For precipitation seasonality (bio15) and precipitation of the warmest quarter (bio18), *M. alternatus* retained relatively broad suitability across moderate-to-high precipitation regimes, while the natural enemies and nematode showed narrower or more scenario-sensitive responses. From an entomological perspective, this supports the expectation that adult emergence, maturation feeding, larval development in wood, and host-tree water stress jointly shape the vector’s realized risk landscape.

The exploratory path model yielded CFI = 1.000, TLI = 1.001, SRMR = 0.002, chi-square(1) = 0.331, *p* = 0.565, AIC = 14,145, and BIC = 14,222 (Figure S2). The reported degrees of freedom were 1. The chi-square test did not reject the specified covariance structure, but the near-perfect fit indices are not evidence of mechanistic adequacy because the model has very few degrees of freedom and was fitted to simplified binary spatial layers.

The largest positive fitted coefficients linked *Pinus* with *B. xylophilus* (2.11), *S. guani* with *D. helophoroides* (1.20), and *D. helophoroides* with *M. alternatus* (1.07). A positive path from *S. guani* to *M. alternatus* was also estimated (0.36). These coefficients indicate strong modeled covariation among host, pathogen, vector, and natural-enemy suitability layers, not necessarily direct beneficial interactions. Because some standardized coefficients exceeded 1, their magnitudes should be interpreted cautiously as features of the fitted covariance structure under categorical inputs and strong covariance among predictors rather than as directly comparable ecological effect sizes.

Weak positive coefficients were estimated from *B. xylophilus* to *D. helophoroides* (0.02) and to *S. guani* (0.05). Two negative conditional coefficients were estimated: *M. alternatus* to *B. xylophilus* (−0.21) and *Pinus* to *D. helophoroides* (−0.11). Their signs describe residual statistical associations after conditioning on the other binary layers. They do not indicate that the vector suppresses the nematode or that *Pinus* suppresses *D. helophoroides*, and no biological or management conclusion is based on these negative coefficients.

## 4. Discussion

### 4.1. Climate-Driven Redistribution of the Pine Wilt Disease Complex

Our results indicate that climate change may alter the geographic configuration of potential suitability across the pine wilt disease complex, with East Asia remaining the dominant area of joint modeled suitability. This statement concerns overlap among model classifications rather than a direct forecast of disease incidence, because correlative species distribution models describe climatic suitability but do not fully represent dispersal, biotic interactions, or establishment processes [51,52]. The *B. xylophilus* projections also identify potentially suitable regions in South America and Africa. We do not infer that the nematode can spread only where *M. alternatus* is suitable, nor that East Asia is the only region at risk. Establishment outside East Asia would depend on introduction pathways, the presence of susceptible local *Pinus* species, and competent local *Monochamus* or other vectors [53,54]. Conversely, the Iberian false-negative region demonstrates that an established population may occur where the model classifies suitability as low or unsuitable, as illustrated by the detection of *B. xylophilus* in Portugal [55]. These boundaries therefore require surveillance and field evidence to be considered alongside the maps.

The projections also revealed strong asymmetry among taxa. *M. alternatus* and *B. xylophilus* were associated with persistent or expanding suitability in East Asia, whereas *S. guani* showed greater scenario dependence and a restricted high-suitability core under stronger warming. Such heterogeneity is expected because climate change acts on distinct biological processes, including beetle emergence, flight, maturation feeding, and oviposition; nematode survival and dispersal stages; parasitoid host searching and development; and host-tree physiological stress [56,57,58]. Consequently, shared exposure to warming does not imply synchronized range shifts or uniform changes in interaction risk.

For *M. alternatus* specifically, the response curves suggest that intermediate temperature seasonality and adequate warm-season moisture may support high suitability. However, the vector’s realized spread is also shaped by active dispersal and host-substrate availability [53,59]. Adults can exploit newly stressed or recently felled trees, move among fragmented patches, and transmit nematodes during maturation feeding before reproduction is complete. These behaviors may allow the vector to respond rapidly to favorable climatic windows and disturbance pulses, potentially outpacing the equilibrium assumption embedded in correlative SDMs [52,59].

### 4.2. Mechanistic Interpretation of Spatial Overlap with Natural Enemies

The high niche and range overlap between *M. alternatus* and *D. helophoroides* should be interpreted as more than broad host tracking. *D. helophoroides* is associated with particular host stages and microhabitats, including late-instar larvae, pupae, and young adults concealed within galleries and pupal chambers. Its females use host-associated frass, tunnel cues, plant volatiles, and visual signals to locate oviposition sites, while newly hatched larvae must contact concealed hosts to establish parasitism [18,19,20]. Therefore, the overlap with *M. alternatus* likely reflects landscapes in which climatic suitability for the natural enemy coincides with the availability of suitable vector stages within woody substrates, rather than a simple map-level coincidence.

This distinction is important for biological inference. Even where *M. alternatus* and *D. helophoroides* overlap, effective suppression depends on local host density, stand structure, wood moisture, oviposition timing, parasitoid abundance, and synchrony between natural-enemy activity and vulnerable vector stages [60]. A high-overlap cell may therefore indicate potential for top-down regulation, but it does not guarantee parasitism or measurable population suppression. Similarly, *S. guani* occupies a broader potential environmental envelope but has a smaller high-suitability core, suggesting that its use as a biological-control agent may require climate-matched strains, carefully timed releases, and local validation under future climate conditions [57].

### 4.3. Interpreting the Conditional M. alternatus-B. xylophilus Coefficient

The negative conditional coefficient between *M. alternatus* and *B. xylophilus* must not be interpreted as biological antagonism. Their known relationship is transmission-mediated: dauer juveniles of *B. xylophilus* are carried by emerging *Monochamus* adults and enter host trees through maturation-feeding or oviposition wounds [12,15,16]. The coefficient instead describes the residual association between two thresholded spatial classifications after the other layers in the fitted equation are held constant, rather than a direct measure of their biological interaction.

With strongly correlated binary predictors, thresholding and conditioning can produce a coefficient whose sign differs from the unadjusted biological relationship. Differences in geographic prevalence, host-layer aggregation, and covariance with the remaining predictors can all contribute to this type of sign reversal or aggregation effect [61,62]. Because the model contains no vector abundance, infection prevalence, nematode load per beetle, transmission events, or temporal ordering, it cannot distinguish among these statistical sources or assign a biological mechanism to the negative sign. The use of SDM-derived binary layers rather than direct observations, together with thresholding, spatial aggregation, and conditioning on correlated predictors, provides a plausible statistical explanation for the counterintuitive coefficient sign; however, the incomplete project archive prevents retrospective sensitivity analysis.

We therefore treat the −0.21 coefficient as a diagnostic warning about the limitations of conditional analysis of thresholded layers. It is retained for transparent reporting of the fitted model, but it is not used as evidence about vector–pathogen biology and should be tested, if pursued, with field data on vector abundance, nematode carriage, tree infection status, and temporal transmission.

### 4.4. Implications for Biological Control of M. alternatus Under Climate Change

The projected overlap between *M. alternatus* and its natural enemies suggests that biological control merits targeted field evaluation within parts of the projected overlap landscape, but any intervention will need to be spatially and phenologically targeted [63]. *D. helophoroides* appears especially relevant as a candidate for field validation in regions where its high-suitability core overlaps expanding *M. alternatus* suitability in eastern China and Japan. Because *D. helophoroides* attacks concealed larval and pupal stages, releases should be timed to the period when host larvae or pupae are present in galleries rather than only to adult flight peaks. Semiochemical tools based on host-associated volatiles or frass cues could improve monitoring or enhance local natural-enemy retention in stands where *M. alternatus* breeding substrates are abundant [18,19,64].

Climate change also alters this timing problem. If warming advances adult emergence or lengthens the maturation-feeding period of *M. alternatus*, control actions that target only late-season larvae may miss the transmission window. Conversely, releases of natural enemies without sufficient host-stage availability may fail even in climatically suitable regions, because temperature can alter parasitoid development, survival, and synchrony with hosts [57]. Adaptive biological control should therefore combine SDM-based hotspot prioritization with field phenology models, degree-day monitoring, and repeated local validation of parasitism and vector suppression [65].

### 4.5. Ecosystem Risk, Resilience, and Management Priorities

The spatial convergence of *B. xylophilus*, *M. alternatus*, *Pinus*, and natural enemies has direct implications for forest resilience. Where host, vector, and pathogen suitability increasingly coincide, pine wilt outbreak risk may rise, with cascading consequences for carbon storage, stand structure, biodiversity, and ecosystem services [58,66,67,68]. Because pine-dominated forests also contribute to climate mitigation and landscape stability, intensified disease pressure could propagate beyond the focal taxa.

At the same time, co-suitability with natural enemies identifies locations where biological-control field studies may be prioritized, not locations where suppression is assured, because realized control depends on host availability, natural-enemy abundance, and temporal synchrony [69]. Priority regions include areas of increasing joint modeled suitability in East Asia, but surveillance should also extend to regions in South America, Africa, and Europe where *B. xylophilus* is modeled as climatically suitable and susceptible hosts, competent local vectors, or introduction pathways may occur. The maps do not establish that *M. alternatus* is the only relevant vector outside East Asia, as several *Monochamus* species can act as potential vectors of the pinewood nematode [8]. Climate-marginal cells also warrant attention because active vector dispersal and movement of infested wood can generate occurrences that are not captured by equilibrium SDMs [52,54].

### 4.6. Limitations and Future Research Directions

Several limitations constrain the interpretation of these results. First, future predictors and suitability maps represent averages across three GCMs. Inter-model dispersion was not retained in the archived outputs; therefore, differences among SSP maps cannot be evaluated against GCM-specific uncertainty. In addition, threshold selection, spatial resolution, and geographic bias in occurrence records can substantially influence binary suitability classifications and their spatial patterns [70,71]. The false-negative classification of known *B. xylophilus* occurrences in Portugal and western Spain further demonstrates that modeled low suitability or unsuitability should not be interpreted as evidence of absence [55].

Second, the *Pinus* layer pools species with distinct climatic niches and differing susceptibility to pine wilt disease. This aggregation may broaden the apparent host envelope and overestimate disease-relevant host availability where resistant or tolerant pine species dominate. Conversely, it may dilute the spatial signal of highly susceptible hosts and underestimate local concentrations of susceptible habitat. The direction and magnitude of this bias are likely to vary geographically; thus, the genus-level layer should be interpreted as broad host availability rather than as a direct measure of disease susceptibility [53].

Third, the exploratory path model is based on thresholded binary layers and cannot quantify abundance, infection prevalence, parasitism, transmission rates, or causal interaction strength. Its fit indices may also appear optimistic because of the simplified structure and only one degree of freedom. In addition, the surviving project archive lacks the complete original pseudo-absence realization, random seed, taxon-specific retained-predictor and VIF tables, numerical binary thresholds, analysis scripts, model objects, and GCM-specific intermediate rasters. These omissions prevent exact computational replication and retrospective quantification of several sources of uncertainty. The study should therefore be interpreted as a screening-level geographic synthesis, consistent with current recommendations for transparent reporting and reproducible SDM workflows [72,73].

Future work should integrate higher-resolution environmental data with standardized multispecies monitoring, vector movement, nematode carriage, host susceptibility, and field estimates of parasitism. Reproducible extensions should archive occurrence filters, pseudo-absence settings and seeds, taxon-specific retained predictors and VIF values, numerical thresholds, scripts, model objects, and GCM-specific rasters. Species-level host layers and independent field validation across predicted hotspots are particularly important. To move from potential co-suitability toward operational forecasts of pine wilt disease, future studies will need process-explicit models that integrate degree-day phenology, dispersal kernels, infection dynamics, and biological-control release schedules [74].

This study provides a screening-level, multispecies view of potential climatic suitability across the pine wilt disease complex. The most defensible result is the concentration of high joint modeled suitability in East Asia, especially southeastern China and Japan, together with wider single-taxon suitability for *B. xylophilus* in parts of the Americas and Africa. These patterns identify priorities for surveillance and field validation; they are not forecasts of disease incidence. The exploratory path model is secondary to the SDM and overlay results, and its negative *M. alternatus–B. xylophilus* coefficient is a conditional feature of thresholded spatial layers rather than evidence of antagonism. Interpretation must also account for the Iberian false-negative region, the genus-level *Pinus* layer, unquantified inter-GCM variability, and incomplete computational archiving.

## Data Availability

The original contributions presented in this study are included in the article/Supplementary Materials. Further inquiries can be directed to the corresponding author.

## Supplementary Materials

The following supporting information can be downloaded at: https://www.mdpi.com/article/10.3390/insects17080819/s1. Figure S1. Analytical workflow of this study. Occurrence records and environmental predictors were used to fit taxon-specific ensemble SDMs. Continuous suitability outputs were evaluated and summarized as suitability classes or thresholded binary layers. These derived layers were subsequently used for niche similarity, geographic range overlap, multispecies co-suitability, response-curve analyses, and an exploratory path analysis. The path analysis used SDM-derived binary classifications rather than direct observations of abundance, infection, parasitism, nematode load, or transmission and therefore does not represent a causal interaction model; Figure S2. Exploratory path model fitted to binary suitability layers for the five focal components. This path analysis is exploratory and non-reproducible from the surviving project archive. Arrow direction represents the specified regression equations and does not indicate causal direction. Blue and red arrows denote positive and negative conditional path coefficients, respectively; they do not indicate facilitation, antagonism, transmission, or biological-control effects. Values beside arrows are conditional path coefficients. Model-fit indices are shown for descriptive purposes only (degrees of freedom = 1). The diagram summarizes conditional broad-scale spatial associations among model classifications, not ecological effect sizes or realized interaction strengths. (a) *Monochamus alternatus*; (b) *Dastarcus helophoroides*; (c) *Scleroderma guani*; (d) *Bursaphelenchus xylophilus*; and (e) *Pinus*; Figure S3. Response curves for the dominant environmental predictors of the five focal components. bio4, temperature seasonality; bio11, mean temperature of the coldest quarter; bio15, precipitation seasonality; bio18, precipitation of the warmest quarter. (a) *Monochamus alternatus*; (b) *Dastarcus helophoroides*; (c) *Scleroderma guani*; (d) *Bursaphelenchus xylophilus*; and (e) *Pinus*; Table S1. Abbreviations used in the manuscript; Table S2. Initial candidate environmental predictors considered before taxon-specific collinearity screening; Table S3. Mean AUC, TSS, and KAPPA values for the five focal components across the candidate modeling approaches and the ensemble model; Table S4. Projected areas of potential geographic distribution for *Monochamus alternatus* under future climate scenarios (×10^4^ km^2^); Table S5. Projected areas of potential geographic distribution for *Dastarcus helophoroides* under future climate scenarios (×10^4^ km^2^); Table S6. Projected areas of potential geographic distribution for *Scleroderma guani* under future climate scenarios (×10^4^ km^2^); Table S7. Projected areas of potential geographic distribution for *Bursaphelenchus xylophilus* under future climate scenarios (×10^4^ km^2^).
