# Supplementary material for: Climate-Driven Changes in Potential Suitability and Spatial Co-Occurrence Across the Pine Wilt Disease Complex
Source: Insects. 2026 Aug 6;17(8):819. doi: 10.3390/insects17080819 (PMC13512961; doi:10.3390/insects17080819)
Supplement: Supplementary file 1 [file insects-17-00819-s001.zip › insects-4452875-supplementary.pdf]

Figures S1–S3 document the analytical workflow, response curves, and the exploratory and non-reproducible path analysis, respectively. Table S1 provides the consolidated abbreviation list requested for rapid reference. Table S2 documents the initial environmental predictor set; Table S3 reports model evaluation; Tables S4–S7 report projected habitat-area summaries.

Supplementary Materials

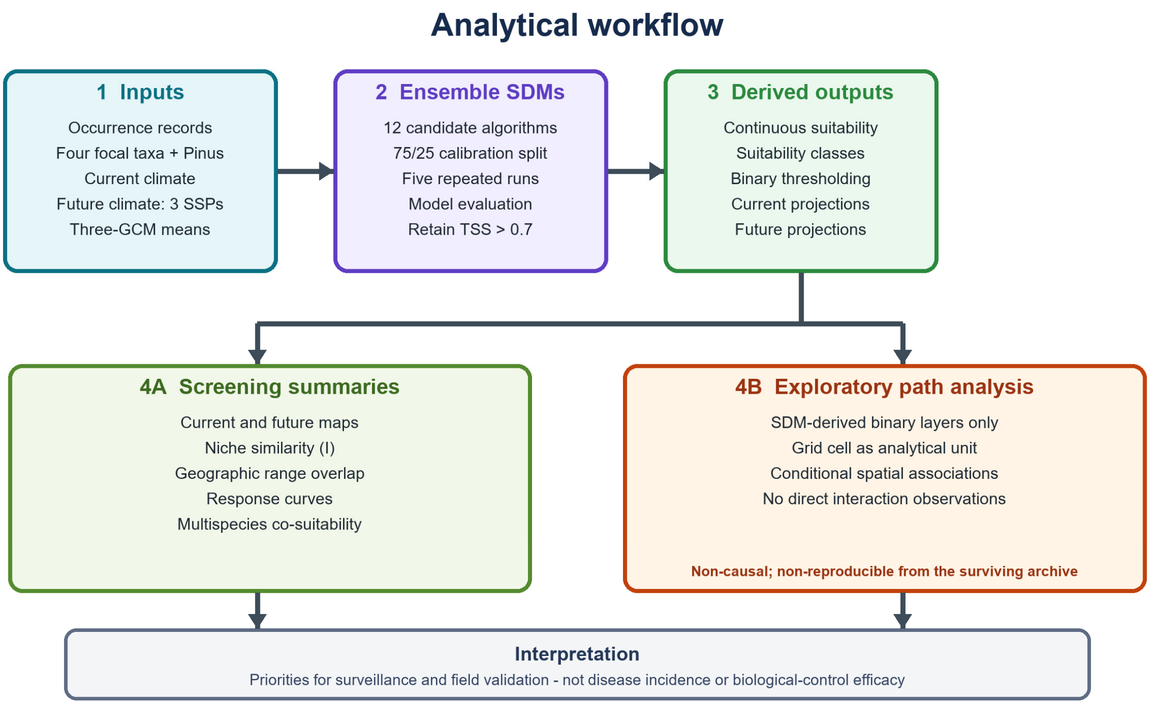

Figure S1. Analytical workflow of this study. Occurrence records and environmental predictors were used to fit taxon-specific ensemble SDMs. Continuous suitability outputs were evaluated and summarized as suitability classes or thresholded binary layers. These derived layers were subsequently used for niche similarity, geographic range overlap, multispecies co-suitability, response-curve analyses, and an exploratory path analysis. The path analysis used SDM-derived binary classifications rather than direct observations of abundance, infection, parasitism, nematode load, or transmission and therefore does not represent a causal interaction model.

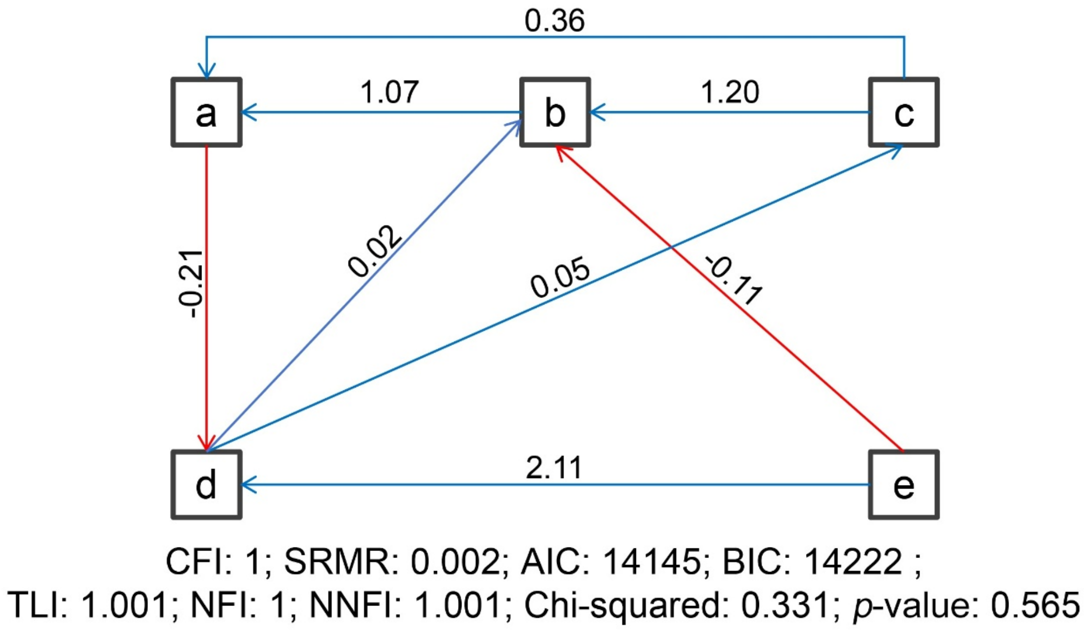

Figure S2. Exploratory path model fitted to binary suitability layers for the five focal components. This path analysis is exploratory and non-reproducible from the surviving project archive. Arrow direction represents the specified regression equations and does not indicate causal direction. Blue and red arrows denote positive and negative conditional path coefficients, respectively; they do not indicate facilitation, antagonism, transmission, or biological-control effects. Values beside arrows are conditional path coefficients. Model-fit indices are shown for descriptive purposes only (degrees of freedom = 1). The diagram summarizes conditional broad-scale spatial associations among model classifications, not ecological effect sizes or realized interaction strengths. (a) *Monochamus alternatus*; (b) *Dastarcus helophoroides*; (c) *Scleroderma guani*; (d) *Bursaphelenchus xylophilus*; and (e) *Pinus*.

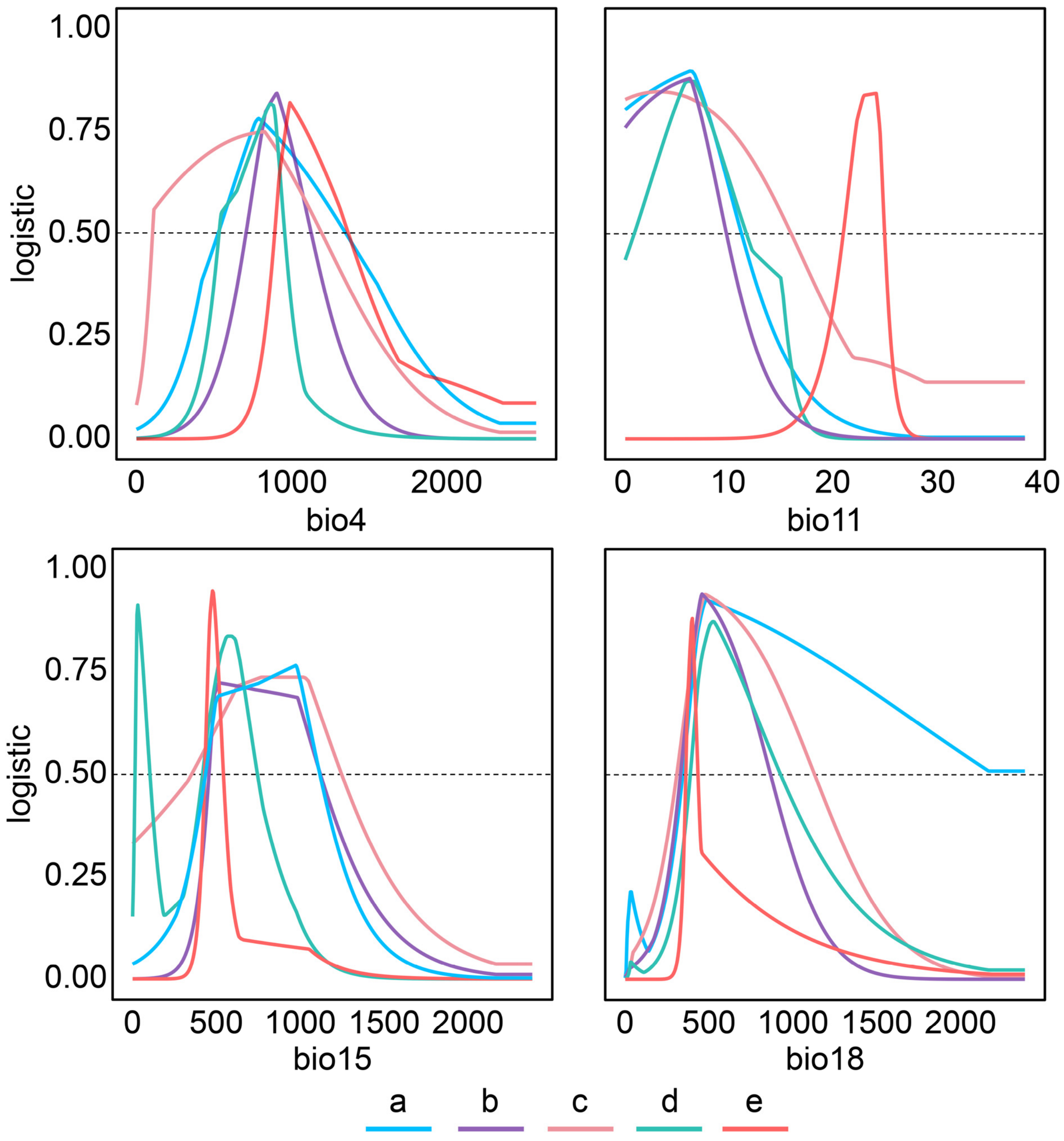

Figure S3. Response curves for the dominant environmental predictors of the five focal components. bio4, temperature seasonality; bio11, mean temperature of the coldest quarter; bio15, precipitation seasonality; bio18, precipitation of the warmest quarter. (a) *Monochamus alternatus*; (b) *Dastarcus helophoroides*; (c) *Scleroderma guani*; (d) *Bursaphelenchus xylophilus*; and (e) *Pinus*.

Table S1. Abbreviations used in the manuscript.

Abbreviation	Meaning
ACCESS-CM2	Australian Community Climate and Earth System Simulator Coupled Model 2
AIC	Akaike information criterion

Abbreviation	Meaning
ANN	Artificial neural network
AUC	Area under the receiver operating characteristic curve
BCC-CSM2-MR	Beijing Climate Center Climate System Model version 2, medium resolution
BIC	Bayesian information criterion
biomod2	R package for ensemble species distribution modelling
CABI	Centre for Agriculture and Biosciences International
CFI	Comparative fit index
CI	Confidence interval
CMCC-ESM2	Euro-Mediterranean Center on Climate Change Earth System Model 2
CTA	Classification tree analysis
EM	Ensemble model
ENMTools	Ecological Niche Modeling Tools
FDA	Flexible discriminant analysis
GAM	Generalized additive model
GBIF	Global Biodiversity Information Facility
GBM	Generalized boosted model
GCM	General circulation model
GLM	Generalized linear model
HFI	Human footprint index
I	Hellinger-based niche similarity index
KAPPA	Cohen's kappa coefficient
lavaan	R package for structural equation modelling
MARS	Multivariate adaptive regression splines
MaxEnt	Maximum entropy model
MAXNET	Maximum entropy model fitted with *maxnet*
R	R statistical computing environment
RF	Random forest
SDM	Species distribution model
SEM	Structural equation model
SRE	Species range envelope
SRMR	Standardized root mean square residual
SSP	Shared Socioeconomic Pathway
TLI	Tucker-Lewis index

Abbreviation	Meaning
TSS	True skill statistic
VIF	Variance inflation factor
XGBoost	Extreme gradient boosting

Table S2. Initial candidate environmental predictors considered before taxon-specific collinearity screening.

Code	Predictor	Source
bio1	Annual Mean Temperature	WorldClim (1970-2000)
bio2	Mean Diurnal Range	WorldClim (1970-2000)
bio3	Isothermality	WorldClim (1970-2000)
bio4	Temperature Seasonality	WorldClim (1970-2000)
bio5	Maximum Temperature of Warmest Month	WorldClim (1970-2000)
bio6	Minimum Temperature of Coldest Month	WorldClim (1970-2000)
bio7	Temperature Annual Range	WorldClim (1970-2000)
bio8	Mean Temperature of Wettest Quarter	WorldClim (1970-2000)
bio9	Mean Temperature of Driest Quarter	WorldClim (1970-2000)
bio10	Mean Temperature of Warmest Quarter	WorldClim (1970-2000)
bio11	Mean Temperature of Coldest Quarter	WorldClim (1970-2000)
bio12	Annual Precipitation	WorldClim (1970-2000)
bio13	Precipitation of Wettest Month	WorldClim (1970-2000)
bio14	Precipitation of Driest Month	WorldClim (1970-2000)
bio15	Precipitation Seasonality	WorldClim (1970-2000)
bio16	Precipitation of Wettest Quarter	WorldClim (1970-2000)
bio17	Precipitation of Driest Quarter	WorldClim (1970-2000)
bio18	Precipitation of Warmest Quarter	WorldClim (1970-2000)
bio19	Precipitation of Coldest Quarter	WorldClim (1970-2000)
elev	Elevation	WorldClim
hfi	Human Footprint Index	SEDAC

Note: This table documents the candidate set; the complete taxon-level retained-variable and VIF table was not preserved in the surviving project archive.

Table S3. Mean AUC, TSS, and KAPPA values for the five focal components across the candidate modeling approaches and the ensemble model.

Species	Score	ANN	CTA	FDA	GAM	GBM	GLM	MARS	MAX ENT	MA XN ET	RF	SRE	XGB OOS T	EM
	AUC	0.66	0.94	0.97	0.98	0.99	0.99	0.98	0.97	0.99	0.78	0.72	0.81	0.99
M. alternatus	TSS	0.49	0.86	0.85	0.87	0.90	0.91	0.89	0.88	0.92	0.76	0.44	0.97	0.91
	KAPPA	0.40	0.82	0.81	0.83	0.88	0.88	0.87	0.90	0.88	0.81	0.48	0.96	0.86
	AUC	0.71	0.97	0.98	0.99	0.69	0.75	0.82	0.98	0.88	0.77	0.97	0.79	0.95
D. helophoroides	TSS	0.49	0.93	0.92	0.93	0.97	0.98	0.97	0.95	0.97	0.69	0.43	0.99	0.97
	KAPPA	0.34	0.83	0.82	0.84	0.92	0.94	0.92	0.97	0.93	0.80	0.52	0.97	0.90
	AUC	0.80	0.90	0.95	0.95	0.98	0.97	0.98	0.85	0.97	0.90	0.67	0.76	0.99
S. guani	TSS	0.63	0.78	0.75	0.76	0.87	0.87	0.69	0.84	0.77	0.34	0.99	0.77	0.91
	KAPPA	0.45	0.57	0.66	0.66	0.75	0.69	0.72	0.79	0.75	0.76	0.27	0.95	0.81
	AUC	0.89	0.90	0.78	0.88	0.91	0.81	0.85	0.90	0.71	0.95	0.81	0.85	0.95
B. xylophilus	TSS	0.68	0.71	0.66	0.76	0.80	0.60	0.66	0.72	0.59	0.71	0.77	0.66	0.80
	KAPPA	0.66	0.78	0.72	0.90	0.65	0.83	0.81	0.77	0.67	0.67	0.90	0.56	0.89
	AUC	0.70	0.61	0.75	0.88	0.81	0.87	0.78	0.90	0.69	0.76	0.93	0.68	0.89
Pinus	TSS	0.45	0.56	0.66	0.61	0.70	0.67	0.59	0.73	0.59	0.66	0.81	0.60	0.80
	KAPPA	0.65	0.78	0.91	0.87	0.66	0.79	0.59	0.88	0.86	0.90	0.59	0.87	0.90

Note: Abbreviations: EM, ensemble model; ANN, artificial neural network; CTA, classification tree analysis; FDA, flexible discriminant analysis; GAM, generalized additive model; GBM, gradient boosting model; GLM, generalized linear model; MARS, multivariate adaptive regression splines; MaxEnt, maximum entropy model; MAXNET, maximum entropy model fitted with MAXNET; RF, random forest; SRE, species range envelope; XGBoost, extreme gradient boosting.

Table S4. Projected areas of potential geographic distribution for *Monochamus alternatus* under future climate scenarios ($\times 10^4$ km^2).

Future scenario	Unsuitable	Low suitability	Moderate suitability	High suitability
2050s-SSP126	10690.83	2924.43	949.16	335.56
2050s-SSP370	10333.77	3076.48	1116.96	372.78

Future scenario	Unsuitable	Low suitability	Moderate suitability	High suitability
2050s-SSP585	10092.54	3172.44	1234.37	400.63
2090s-SSP126	10500.92	3059.73	978.42	360.91
2090s-SSP370	9290.87	3607.99	1537.95	463.17
2090s-SSP585	8881.19	3862.61	1679.63	476.55

Table S5. Projected areas of potential geographic distribution for *Dastarcus helophoroides* under future climate scenarios (×10^4 km^2).

Future scenario	Unsuitable	Low suitability	Moderate suitability	High suitability
2050s-SSP126	12395.64	2009.85	317.67	176.82
2050s-SSP370	12363.10	2007.08	333.61	196.19
2050s-SSP585	12236.73	2102.32	363.37	197.56
2090s-SSP126	12559.82	1843.35	332.35	164.46
2090s-SSP370	11636.03	2614.76	446.92	202.27
2090s-SSP585	10712.74	3465.26	511.81	210.17

Table S6. Projected areas of potential geographic distribution for *Scleroderma guani* under future climate scenarios (x 10^4 km^2).

Future scenario	Unsuitable	Low suitability	Moderate suitability	High suitability
2050s-SSP126	5.15	13187.40	1703.93	3.49
2050s-SSP370	1.18	10780.32	4064.05	54.43
2050s-SSP585	2.15	12224.56	2620.97	52.29
2090s-SSP126	7.34	12872.55	2009.76	10.33
2090s-SSP370	11.37	10784.46	4068.58	45.57
2090s-SSP585	1.48	12662.89	2233.70	1.91

Table S7. Projected areas of potential geographic distribution for *Bursaphelenchus xylophilus* under future climate scenarios (×10^4 km^2).

Future scenario	Unsuitable	Low suitability	Moderate suitability	High suitability
2050s-SSP126	11754.82	2035.27	592.86	441.49
2050s-SSP370	11647.01	2118.84	621.07	437.52

Future scenario	Unsuitable	Low suitability	Moderate suitability	High suitability
2050s-SSP585	11009.34	2652.81	677.81	484.48
2090s-SSP126	11315.80	2390.64	660.99	457.00
2090s-SSP370	11046.88	2667.22	666.50	445.57
2090s-SSP585	10928.34	2713.81	797.32	384.97
